# A public health study on the participation mechanism of social capital in the governance of public sports space in dilapidated urban communities – a case study of Changsha City, Hunan Province

**DOI:** 10.3389/fpubh.2023.1100137

**Published:** 2023-07-31

**Authors:** Li Xinze, Zhang Xiaoyi, He Qiao

**Affiliations:** ^1^School of Humanities, Beijing Sport University, Beijing, China; ^2^School of Strength and Conditioning Training, Beijing Sport University, Beijing, China

**Keywords:** dilapidated communities, public sports spaces, social capital, community governance, sustainable development

## Abstract

**Introduction:**

China’s aging population, mobile population, low-income families, and other vulnerable groups congregate in dilapidated urban communities serving as public health spaces. As a result, managing public sports spaces in aging urban areas is a significant public health project in China, an essential strategy for raising residents’ quality of life, and a significant effort to support the active aging of the older adult.

**Methods:**

The study used mathematical and statistical techniques, questionnaires, and logical deduction to conduct a public health study on the participation mechanism of social capital in the governance of public sports spaces in dilapidated urban communities. It chose 11 old Changsha, Hunan Province, communities as the research objects.

**Results:**

Personal social capital was found to boost the availability of public sports spaces in older populations through social connections. Collective social capital improves the availability of public sports spaces in aging populations through social trust and stabilizes the order of public sports spaces in aging communities through social involvement.

**Discussion:**

To improve the governance efficiency of public sports spaces in aging urban communities, the study aims to actively mobilize and accumulate social capital through cultivating the public spirit, reshaping the concept of sports governance, appropriately decentralizing and empowering, strengthening sports governance structures, enhancing communication and collaboration, and building sports governance. This is essential for China to fully implement the policies of active aging, a healthy China, and creating a community for global public health.

## 1. Introduction

The main objective of China’s innovative public health governance is to “build a public health community in which everyone is responsible and does his or her part,” with a particular emphasis on encouraging the peaceful coexistence of various governance issues (1). The burden on public health governance is also growing daily due to multiple emergencies and diverse interests. Urban communities’ management of public sports spaces during the New Crown Pandemic exposed issues such as low public involvement, a lack of social capital, and professional scarcity. The community has steadily developed into a significant carrier of residents’ numerous needs for sports space, living environment, social contact, emotional connection, and interest expression, along with the ongoing decline of public health governance in China (2). Therefore, integrating resources, collaborating, mobilizing social capital, increasing resident engagement, and creating wider social cohesion is crucial to the governance of public sports spaces in China’s metropolitan communities.

The idea of community control of public sports spaces comes from the notion of good governance, and it is through the harmonious coexistence of the state and society that the goal of maximizing the positive effects on public health is attained (3). With the combined efforts of numerous network systems, such as governmental organizations, social sports organizations, and residents’ autonomous organizations, the governance of community public sports spaces also introduces the concept of governance into the community sports field and realizes the sustainable development of public health governance (4). A challenging issue that needs to be resolved in the governance of community public sports spaces is how to strike a balance between the public health interests of various subjects. This is seen from the perspective of the subjects of governance. China’s public sports space is governed under three primary models: market-led, government-led, and socially autonomous. These models primarily focus on introducing and integrating social capital in the community (5). The purpose of governing community public sports spaces has changed from a single social function to a comprehensive public health role that includes social, economic, and ecological factors. In micro-public health governance areas like community sports environment maintenance and sports atmosphere creation, public participation can frequently produce better results; however, when it comes to complex and comprehensive public health governance issues, the government still needs to play a leading role. Community administration of public sports spaces has to regularly include new innovative components, depending on the governance stage (6). The administration of community public sports space has steadily included digital components and supported the optimization of public health grid management and governance principles from changes in lifestyles and attitudes, along with the broad application of “Internet+” technology (7). In addition, a gradual trend toward resilience in community public sports space governance has emerged in response to the significant new crown epidemic outbreak, advocating that multiple subjects take on governance responsibilities and create a collaborative, shared, and co-constructed public health community (8).

The management of public sports spaces in local communities is significantly influenced by social capital. Regarding categorizing social capital, there are two viewpoints: personal and collective. The former emphasizes how people and community topics are related, while the latter emphasizes the synergistic processes connecting various subjects. According to the concept of social capital’s involvement in connectedness, bridging, and bonding, public health resources can be transformed more easily inside groups, between groups, and between groups and external relationship networks (9). The core governance principles in community public sports spaces are reciprocity, trust, cooperation, and regulation. There is a secure fit between this and the manifestation of social capital in terms of the relationship between social capital and the effectiveness of governance in community public sports spaces (10). For instance, social capital improves the administration of public sports spaces in a community through relational and structural embedding.

Urban communities are increasingly moving from “unitary community spaces” to “stratified community spaces” as we go from a planned economy to a market economy. The middle and privileged classes now live in developing commercial housing developments and villa residential districts with high-quality infrastructure, a beautiful setting, and supportive services (11). On the other hand, due to considerations like development expenses, the price of urban land, and the expenditures associated with destruction during the government’s planning and capital operation phases, dilapidated communities have become the least expensive space in the city. Traditional communities and unified public housing blocks are two examples (12). The Guidance on Comprehensively Promoting the Renovation of Dilapidated urban communities defines dilapidated urban communities as residential areas constructed before 2000 with lower construction standards, aging infrastructure, inadequate supporting spaces, and no long-term management mechanism. As a result, dilapidated communities progressively evolve into places where low-income families, the dilapidated, the migratory population, and other vulnerable groups assemble (13). China’s historic villages are more significant than 3 billion m2, and 39,000 will be renovated in 2020 alone, affecting around 7 million people (14). The massive new crown epidemic outbreak has increased public awareness of public health and the value of public sports spaces. However, the administration of public sports spaces in dilapidated communities still needs to be addressed due to the challenges of an inadequate supply of sports facilities, low utilization of existing sports facilities, unsustainable public health resources, and insufficient sports space for the general convenience of the public. To increase the efficiency of governance of public sports spaces in dilapidated communities and advance the deeper integration of aging and public health development, the nation intends to mobilize and accumulate social capital through resident-led activities, community consultation, government support, and multi-participation.

**Figure 1:**
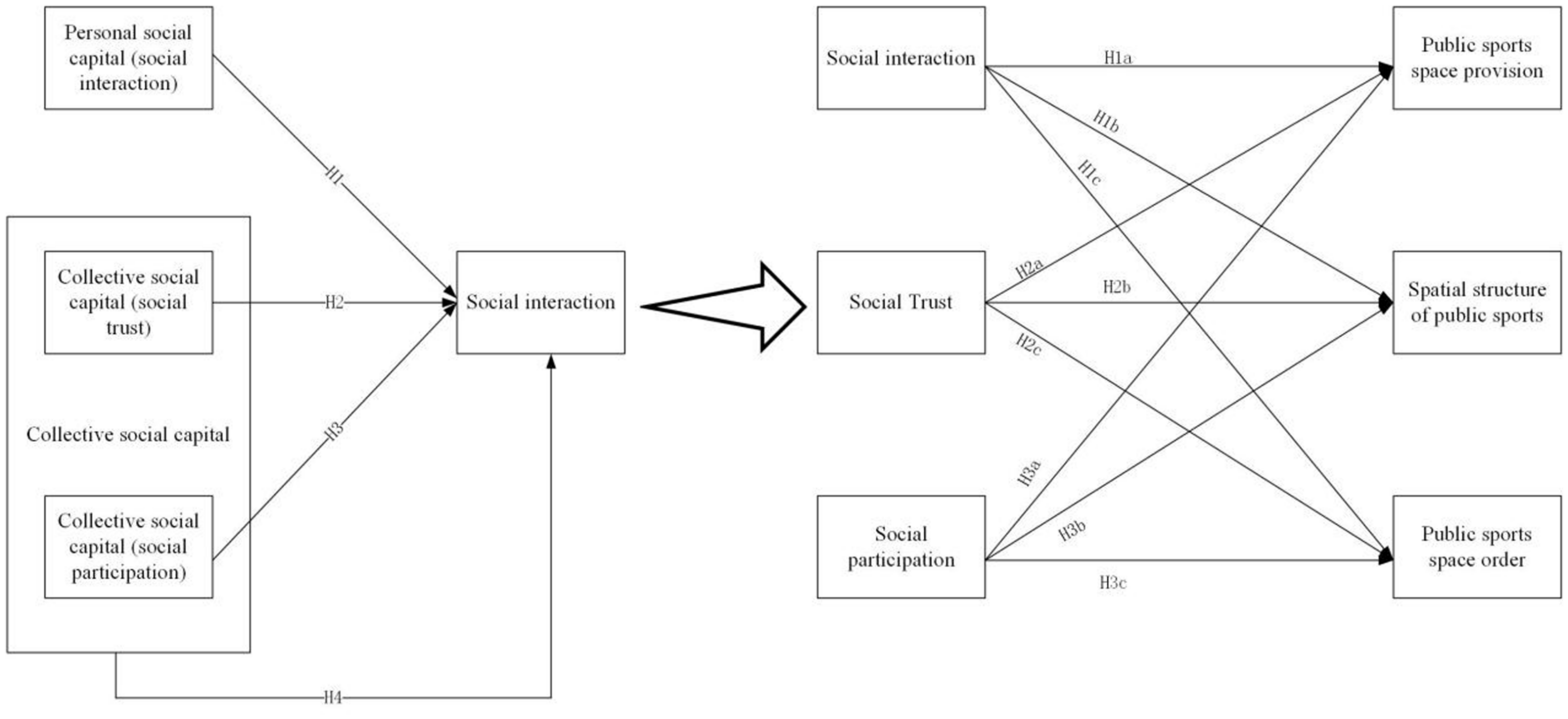
Theoretical model of social capital participation mechanism in the governance of public sports space in dilapidated urban communities.

In conclusion, little research has been done on the influence of social capital on the management of public sports spaces in dilapidated communities. Based on this, this study examines the forms and measurement of social capital, utilizes it as a theoretical framework, and examines social capital’s involvement mechanisms in managing public sports spaces in dilapidated urban populations. The path mechanism for building social capital is refined to create a public health community and strengthen the community’s efforts to promote the health of underprivileged groups (see Figure 1).

## 2. Literature review and research hypothesis

### 2.1. Conceptual interpretation and measurement of social capital

Since Bourdieu (15) and Coleman (16) first proposed the idea of social capital in the 1980s, various fields have favored it because of its potent explanatory power. It has since developed into a crucial theoretical idea in social science research and is frequently used in fields like sociology, economics, management, sports, and public health research. Social capital’s ability to explain phenomena is primarily due to the concept’s more open definition, which allows for several interpretations across research. The definitions and theories of social capital have been thoroughly reviewed, and it is maintained that the disagreements and academic discussions over these topics are primarily the result of variations in the degrees of analysis researchers utilize. The researcher separates social capital into two categories (17–19), personal and collective social capital, and offers a thorough evaluation of their definitions and measuring techniques. This is done following the degrees of study of social capital.

Portes (20) and Linnan (21) are exemplary academics who have studied personal social capital. Academics today frequently use Linnan’s concept of personal social capital, which says that it is a resource entrenched in social networks and social interactions that may be accessed or mobilized for specific purposes. The analysis of personal social capital at the personal level concentrates on three aspects: investment in social capital, access to and mobilization of social capital, and returns to social capital, i.e., how personals invest in social relationships and social networks, how they access and use the resources embedded in their personal social relationships and social networks, and how they benefit from instrumental action and maintain the advantages of effective action. The majority of measurement techniques are normative and locative. A person’s social network, such as a “New Year’s Eve network” or a “restaurant network,” is measured using the nomination technique (22), which also evaluates factors including network size, network composition, network heterogeneity, and network topology. The positioning approach primarily assesses an individual’s public health resources, and socioeconomic class and status impact the depth and breadth of those resources. For instance, the stock of social capital is predicted when the quality of public health resources is mapped by employment status (23).

Putnam (24), a pioneer in the study of collective social capital, defines it as “social organization traits, such as trust, norms, and social networks, that enhance the efficiency of society by fostering cooperative behavior.” Collective social capital is “an internal social capital or public good that, in addition to macro-level intra-group social bonds and mutual trust, also includes how groups are formed to facilitate collective action and develop resources,” according to some academics (25). There is no standardized metric for collective social capital at this time. Putnam contends that the critical indicators for foreign nations are social trust, social norms, and reciprocal cooperation. In contrast (26), Woolcock contends that there should be at least four indicators: social networks, social connections, and social norms (27). Zhang Xiaoli views indicators like cooperation and reciprocity, social involvement, and social trust as critical to gauging collective social capital (28); Wu Bao uses emotional and cognitive trust as indicators in the domestic environment (29).

Social capital is primarily expressed through social engagement, participation, and trust. This essay examines the role of social capital as a facilitator of social structures at the personal and collective levels, emphasizing social capital in the governance of community public sports spaces. This essay summarizes the opinions of most academics, who believe that social capital is a characteristic of social structure, a “horizontal link” of stakeholders, and a means through which government may be made more effective through cooperation and communication (30–33). Therefore, the study of the administration of public sports spaces in dilapidated urban communities is ideally suited for studying social capital, a particular resource inherent in the social structure capable of enhancing social efficiency through mutual coordination.

### 2.2. Public health mechanism of social capital participation in the governance of public sports space in dilapidated communities

Early in the twenty-first century, the social capital theory was brought to China by scholars who used it in community development and public health governance. The social capital theory presents a fresh viewpoint on local government. The social capital that underpins a community’s social networks, social interactions, social cohesiveness, and social trust are all great tools for enhancing the efficiency of a community’s governance. Putnam contends that community social capital is the “lubricant” that keeps communities functioning and is essential to their development (34). According to empirical research, communities with high social capital tend to be critical players in fostering community growth, boosting governmental efficiency, and raising resident well-being. As an illustration, the northern regions of Italy have a long history of great public engagement and high efficacy in grassroots administration, but the southern areas have struggled with poor participation and low effectiveness due to a lack of social capital (35). Some academics contend that social capital is crucial and valuable for lifting communities out of poverty and can considerably enhance the general health of the local populace (36, 37).

#### 2.2.1. Public health mechanism of personal social capital participation in the governance of public sports space in dilapidated communities

Based on the Chinese experience, there is an expanding body of study on personal social capital and the administration of public sports spaces in communities. According to some academics (38, 39), a resident’s social capital is significant in determining how involved they are in managing local public sports spaces. It aids in enhancing interpersonal skills and emotional relationships among community members, thereby developing a consistent pattern of cooperation. Some academics contend that it is challenging to successfully enhance the governance of public sports spaces in communities because of the rustiness of inter-individual connections (40, 41). Therefore, the essential elements in developing a “public health community” are removing social barriers between residents of dilapidated towns, fully utilizing the sports resources inside dilapidated communities, and actively mobilizing personal social capital. According to Pan Zechuan (42), frequent social interactions among dilapidated community members will result in the formation of a stable social group, effective participation in public health governance under widespread social norms, and active mobilization of personal social capital, ultimately increasing the effectiveness of the provision of public sports spaces. In Fang Yachen’s opinion (43), personal social capital contributes positively to the spatial organization of community sports. In large part, the geographical qualities of the social structure are reflected in the spatial organization of public sports, and the degree to which this organization is perfected reflects the stock of personal social capital. In other words, community members’ social interaction behaviors encourage the development of their social capital and the rejuvenation of the physical environment for public recreation. Additionally, the psychological desire for social connection among community members creates a psychological space for the mobilization of personal social capital, improving residents’ feelings of community identification and belonging and bolstering the stability of the public sports space order (44). Based on this, the following theoretical hypothesis is proposed for this study:

*H1*: Personal social capital has a positive contribution to the governance of dilapidated public sports spaces.

*H1a*: Social interaction has a positive contribution to the improvement of public sports space supply in dilapidated communities.

*H1b*: Social interaction has a positive contribution to the optimization of public sports space structure in dilapidated communities.

*H1c*: Social interaction has a positive contribution to the stability of public sports space order in dilapidated communities.

#### 2.2.2. Public health mechanism of collective social capital participation in the governance of public sports space in dilapidated communities

The efficient governance of dilapidated communities is a natural fit for collective social capital. The government, local inhabitants, and social forces must work together to control public sports spaces in dilapidated communities. They also need to mobilize and build social capital together actively. Collective social capital is public property, and the social ties it fosters through social networks enable the integration of resources for public health and help to increase the efficiency of public health governance. On the one hand, social trust, a crucial component of collective social capital, contributes to the simplification of interactions and the reduction of transaction costs, improving the quality of the availability of public sports space. Wu Yufeng asserts that the social trust may foster intergenerational collaboration among community members, improve the flow of resources for public health, efficiently realize data exchange, and benefit the optimization of the physical layout of public sports spaces (45). According to Ng Lin (46), residents of communities with higher levels of social trust are more likely to be able to understand and communicate with one another, support and care for marginalized groups in need, to actively work with grassroots sports workers in the community to improve governance and to follow the rules of the community public sports space actively. This leads to achieving effective governance of the community’s public sports space.

On the other hand, social interaction is a crucial means of strengthening the bonds between neighbors and creating a community’s overall social capital, including norms of behavior and shared values. Some academics contend that social involvement significantly enhances community citizens’ involvement in public sports activities, enhancing the standard of public sports spaces (47, 48). According to Chen Yusheng (49), social participation is a hierarchy of clusters, and famous clusters adhere to the “spatial participation – activity participation – organizational participation – political participation” spatial structure model. In other words, increasing social engagement will encourage the renovation of public sports’ physical landscape. Based on the 2018 China Labour Force Dynamics Survey, Zhang Dong developed a thorough case for controlling public sports spaces in communities (50). He contends that social engagement is a type of belief and behavior that intentionally observes and upholds social order. People’s actions are influenced by their social environment, which causes them to act in ways consistent with the social group. In other words, the more socially engaged the residents of the dilapidated community area are, the more they may support the development and civil governance of the spatial order of public sports, speeding up the process of creating a community focused on public health. Based on this, the following theoretical hypothesis is proposed for this study:

*H2*: Collective social capital (social trust) has a positive contribution to the governance of public sports spaces in dilapidated communities.

*H2a*: Social trust has a positive contribution to the improvement of public sports space supply in dilapidated communities.

*H2b*: Social trust has a positive contribution to the optimization of public sports space structure in dilapidated communities.

*H2c*: Social trust has a positive contribution to the stability of public sports space order in dilapidated communities.

*H3*: Collective social capital (social participation) has a positive contribution to the governance of public sport spaces in dilapidated communities.

*H3a*: Social participation has a positive contribution to the improvement of public sports space supply in dilapidated communities.

*H3b*: Social participation has a positive contribution to the optimization of public sports space structure in dilapidated communities.

*H3c*: Social participation has a positive contribution to the stability of public sports space order in dilapidated communities.

*H4*: Collective social capital has a positive contribution to the governance of public sports spaces in dilapidated communities.

In conclusion, public relations research on the connection between social capital and local government control over public sporting spaces is continually being updated. Based on the current literature, this paper makes the case that several viewpoints merit additional investigation and study. First off, the community has gained a certain degree of “immunity” from various risks and challenges by banding together and sharing safety and security, which is vital for the long-term growth of public health governance. Secondly, the outbreak of the new crown epidemic has increased the probability of public health risk events much more than in the past. Second, home segregation not only encourages community residents to use the Internet more frequently and to disseminate public health resources better, but it also boosts sports participation among a variety of subjects and stimulates community residents’ demand for public health, which is helpful for the high-quality growth of community governance of public sports space. Third, analyses of how social capital is measured in dilapidated communities and how public health is related to the management of public sports spaces in dilapidated communities are uncommon and often consist of descriptive case studies or generalizations of local policy experiences. Residents of China’s dilapidated communities suffer from issues like aging, low literacy, and low income, which tend to make residents’ participation in the governance of public sports spaces show weak participation awareness, poor participation channels, and low participation levels in the governance conundrum, creating a vicious cycle of public health governance in China (51). Dilapidated communities’ social capital formations are unique in several ways, including how their social networks are connected, how confident trust is formed, and how their governance systems are organized. Therefore, it is essential to investigate the public health connection between social capital and the administration of public sports spaces in dilapidated communities.

## 3. Research methodology

### 3.1. Questionnaire design

To gain a thorough understanding of the public health relationship between social capital involvement mechanisms and the administration of public sports spaces in urban dilapidated communities. To design the questionnaire with the features of dilapidated communities in China, this paper covers a wide range of domestic and foreign literature on social capital and public sports space regulation. This review is based on familiarity with the significance of the study.

#### 3.1.1. Social capital questionnaire

Three questions (A1–A3) at the level of social interaction were used to create the concept of personal social capital (52). Higher scores on the 5-point Likert scale—used in the study—were associated with more personal social capital. Where 0.83 is Cronbach’s coefficient (social interaction). Six questions from the social trust (B1–B3) and social involvement components comprise the collective social capital (C1–C3). Higher ratings on the 5-point Likert scale employed in the study denoted a more incredible pool of Collective social capital. The Cronbach coefficients of social trust and social participation are 0.714 and 0.921, respectively.

#### 3.1.2. Governance questionnaire for public sports spaces in dilapidated communities

Liang Chin-scale Chiu’s reforming public spaces for community sports is the primary inspiration for the questionnaire for the governance of public sport space in dilapidated communities (53). The three dimensions of public sports space provision (D1–D3), spatial structure of public sports (E1–E3), and general sports space order (F1–F3) were combined to create a total of nine questions. More significant responses on a Likert scale of 5 indicated higher-quality renovations of public sports spaces in dilapidated towns. The general sports space provision, structure, and Cronbach coefficients were 0.883, 0.755, and 0.70, respectively (public sports space order).

### 3.2. Data sources

On the one hand, Changsha’s rehabilitation of its dilapidated communities is beginning to take shape. One hundred twenty-three historic villages are being renovated, totaling 716 homes, 20,278 people, and 1.72 million square meters of construction space. On the other hand, there is a benchmarking effect from the management of public sports spaces in Changsha’s dilapidated communities. Therefore, the research on the mechanism underlying the function of social capital in public sports spaces in historic communities has significant model implications for the administration of public sports spaces in historic communities in other cities.

The study area and research object in this work are 11 established dilapidated communities in Changsha’s Yue Lu District that date back to 2020. The subject group randomly chose 32 families in each community from the list of data the community committees provided to conduct a paper questionnaire survey and distribute consumer vouchers. To ensure the sampling’s scientific, average, and random quality, the subject group created a list of sampled households, which contained the street, community, and dwelling door numbers. Additionally, the research assistant assisted them in filling out the surveys using a question-and-response format, considering the age distribution of the residents in some dilapidated areas. A total of 332 of the 352 questionnaires that were distributed were returned. By removing questions with significant missing data and discrepancies, 297 (89.5%) valid questionnaires were collected. Table 1 displays the demographic details of the entire survey sample. It is important to note that the analysis of the study’s data includes control variables such as gender (54), age (55), education level (56), and frequency of weekly physical exercise (57).

**Table 1:** Table of demographic characteristics of the overall survey sample.

Indicators	Options	Percentage/%
Gender	Male	46.3
	Female	53.7
Age	≤30	13.8
	31< and ≤40	16.1
	41< and ≤50	20.6
	51< and ≤60	28.1
	≥61	21.4
Education level	Primary school and below	42.6
	Junior High School	28.9
	High School	19.7
	University and above	8.8
Frequency of physical activity per week	≤2	48.6
	3≤ and ≤4	27.9
	5≤ and ≤6	17.1
	=7	6.4

Before participating in the study, each personal provided informed consent. Jimei University’s ethics committee authorized the protocol (JMU202204030), and the study was carried out in compliance with the Declaration of Helsinki.

### 3.3. Homoscedasticity variance test

Harman’s single-factor test was used, and the main component factors were evaluated for all of the test items in the questionnaire using spss26.0 to reduce common method bias in the data. The resulting eigenroot values were more significant than 1, with a total variance of 73.057% for the six components and a clash of 20.185% (50%) for the first factor. In conclusion, it does not exhibit the homogeneous variance conundrum, and the common variance’s deviation is within the theoretically permitted range and complies with the statistical criteria.

## 4. Study results and analysis

### 4.1. Correlation analysis

Both with and without adjusting for demographic factors, as shown in Table 2, there was a significant positive association between the two variables. Demographic factors were therefore left out of the follow-up analysis for this study.

**Table 2:** Results of correlation analysis of key variables.

	Social interaction	Social trust	Social participation	Public sports space provision	Spatial structure of public sports	Public sports space order
Social interaction	1	0.47^**^	0.31^**^	0.51^**^	0.12^*^	0.22^**^
Social Trust	0.43^**^	1	0.57^*^	0.11^*^	0.29^**^	0.24^**^
Social participation	0.33^**^	0.59^***^	1	0.08^*^	0.25^*^	0.23^**^
Public sports space provision	0.56^*^	0.28^**^	0.09^*^	1	0.34^***^	0.58^***^
Spatial structure of public sports	0.14^*^	0.34^**^	0.42^*^	0.31^**^	1	0.66^***^
Public sports space order	0.11^**^	0.25^**^	0.36^**^	0.52^**^	0.68^***^	1

Above “1” is the correlation coefficient between two variables after controlling for demographic variables, below “1” is the correlation coefficient between two variables before controlling for demographic variables; ***p < 0.001, **p < 0.01, and *p < 0.05.

### 4.2. Results of the reliability test

Spss26.0 and Amos26.0 were used to examine the dependability of standard loadings, C.R. reliability, Cronbach’s, and AVE values (see Table 3). The C.R. combination reliability ranged from 0.8222 to 0.9462, indicating good internal consistency and reliability among the latent variables; the AVE variance extracts were all greater than 0.6, indicating that the questionnaire had good convergent validity; the standard loadings were all greater than 0.6, indicating that the questionnaire had good convergent validity; and the standard loadings were all greater than 0.6, indicating that the questionnaire had the value of *p* was less than 0.01 (significant), suggesting that the questionnaire has solid structural validity.

**Table 3:** Factor analysis and reliability test table.

Latent variables	Operational definition of an observation variable	Standard load	Cronbach’s α	CR	AVE
Personal social capital	A1: Do you often speak on the phone, WeChat, or QQ with members of social organisations?	0.745^***^	0.83	0.8564	0.6662
	A2: Do you often engage with Weibo and Shakeology users who are part of social groups?	0.86^***^			
	A3: Do you often publish on social networking sites run by charitable organisations?	0.839^***^			
Collective social capital	B1: Do you think social organisation leaders can be trusted to be truthful and dependable in their statements?	0.76^***^	0.714	0.8222	0.6065
	B2: Do you think that social group members can be trusted to keep their commitments to one another?	0.796^***^			
	B3: How much do you believe your buddies in social groups?	0.78^***^			
	C1: Do members of social organisations extend greetings to one another upon meeting?	0.936^***^	0.921	0.9462	0.8543
	C2: Do social group members generally communicate well and work well together?	0.943^***^			
	C3: Do you often get assistance from people in social organisations?	0.893^***^			
Public sports space provision	D1: Are you happy with the community’s provision of scientific fitness guidance?	0.875^***^	0.883	0.9107	0.7727
	D2: Do you have enough public sports spaces in your town for sporting events?	0.885^***^			
	D3: Are you happy with the upkeep of the public sports spaces in your neighbourhood?	0.877^***^			
Spatial structure of public sports	E1: Do you still find the design of public sports spaces in your town to be satisfactory?	0.813^***^	0.755	0.8567	0.6668
	E2: Do you believe there are more public sports spaces in your neighbourhood?	0.878^***^			
	E3: Do you believe that the community’s public sports spaces are becoming more functional?	0.754^***^			
Public sports space order	F1: I absolutely like the community’s sports setting and vibe.	0.842^***^	0.705	0.8293	0.6227
	F2: Do local residents often use the public sports spaces?	0.873^***^			
	F3: Do you support real estate management firms that oversee public sports spaces in your neighbourhood?	0.63^***^			

***p < 0.001.

### 4.3. Results of the suitability test

CFI = 0.982 (>0.9), NFI = 0.934 (>0.9), TLI = 0.977 (>0.9), three of which are value-added fitness tests; PCFI = 0.777 (>0.5), PNFI = 0.739 (>0.5), two of which are *x*^2^ = 162.362 (the smaller, the better), *x*^2^/DF = 1.342 (<3.0), RMSEA = 0.034 (<0.08), and GFI = 0.982 (>0.9), all four of which are absolute fitness tests (58) (see Table 4). In summary, the value-added fitness test index, the parsimony fitness test index, and the whole fitness test index, all three fitted values, are within the valid range of the recommended values, proving that the theoretical model of this study has good fitness.

**Table 4:** Theoretical model fitness test table.

Adaptation indicators	Recommended value	Fitted values
*χ*2	The smaller the better	162.362
*χ*2/DF	<3.0	1.342
RMSEA	<0.08	0.034
NFI	>0.9	0.934
IFI	>0.9	0.982
CFI	>0.9	0.982
GFI	>0.9	0.944
AGFI	>0.8	0.92
TLI	>0.9	0.977
PNFI	>0.5	0.739
PCFI	>0.5	0.777

### 4.4. Path coefficients and hypothesis testing

The results of the path relationship test showed (as in Table 5). “Personal social capital (social interaction) → public sports space provision,” “Personal social capital (social interaction) → spatial structure of public sports,” and “Personal social capital (social interaction) → public sports space order.” The standardized factor loadings for “personal social capital (social interaction) → public sport spatial order” were 0.584 (*p* < 0.001), −0.043 (*p* = 0.705), and −0.020 (*p* = 0.755), respectively; and the *T* values were 4.729, −0.378, and −0.312, respectively. Among them, only “personal social capital (social interaction) → public sports space provision” with |*T*| > 1.96 has a positive effect, H1a is valid, H1b and H1c are not. In conclusion, the standardized factor loadings for “personal social capital → governance of public sport space” were 0.694 (*p* < 0.001), with a *t*-value of 5.967 (|*T*| > 1.96), which has a positive effect and H1 holds.

**Table 5:** Table of results of structural equation model path tests.

Path relationships	Standardised factor loadings	*T*	*P*	Hypothesis testing
Personal social capital (Social interaction) → public sports space provision	0.584	4.729	^***^	Support
Personal social capital (Social interaction) → Spatial structure of public sport	−0.043	−0.378	0.705	Not supported
Personal social capital (Social interaction) → public sports space order	−0.02	−0.312	0.755	Not supported
Personal social capital → governance of public sport space	0.694	5.967	^***^	Support
Collective social capital (Social Trust) → public sports space provision	0.453	4.074	^***^	Support
Collective social capital (Social Trust) → Spatial structure of public sport	0.004	0.042	0.967	Not supported
Collective social capital (Social Trust) → public sports space order	−0.082	−1.39	0.164	Not supported
Collective social capital (Social Trust) → governance of public sport space	0.449	4.059	^***^	Support
Collective social capital (Social participation) → public sports space provision	0.064	1.266	0.205	Not supported
Collective social capital (Social participation) → Spatial structure of public sport	0.013	0.276	0.728	Not supported
Collective social capital (Social participation) → Public sports space order	0.128	2.438	^*^	Support
Collective social capital (Social participation) → governance of public sport space	0.024	0.409	0.682	Not supported
Collective social capital → governance of public sport space	0.123	2.164	^*^	Support

***p < 0.001, **p < 0.01, and *p < 0.05.

“Collective social capital (social trust) → public sports space provision,” “collective social capital (social trust) → spatial structure of public sports,” and “collective social capital (social trust) → public sports space order.” The standardized factor loadings for “collective social capital (social trust) → public sports space provision,” “collective social capital (social trust) → spatial structure of public sports,” and “collective social capital (social trust) → public sports space order” were 0.453 (*p* < 0.001), 0.004 (*p* = 0.967), and − 0.082 (*p* = 0.164), respectively; and the *T* values were 1.981, 0.042, and −1.390, respectively. Among them, only “collective social capital (social trust) → public sports space provision” has a positive effect with |*T*| > 1.96, and H2a is valid, while H1b and H1c are not. The standardized factor loading for “collective social capital (social trust) → governance of public sport space” was 0.449 (*p* < 0.001), with a *t*-value of 4.059 (|*T*| > 1.96), which has a positive effect and H2 holds.

**Figure 2:**
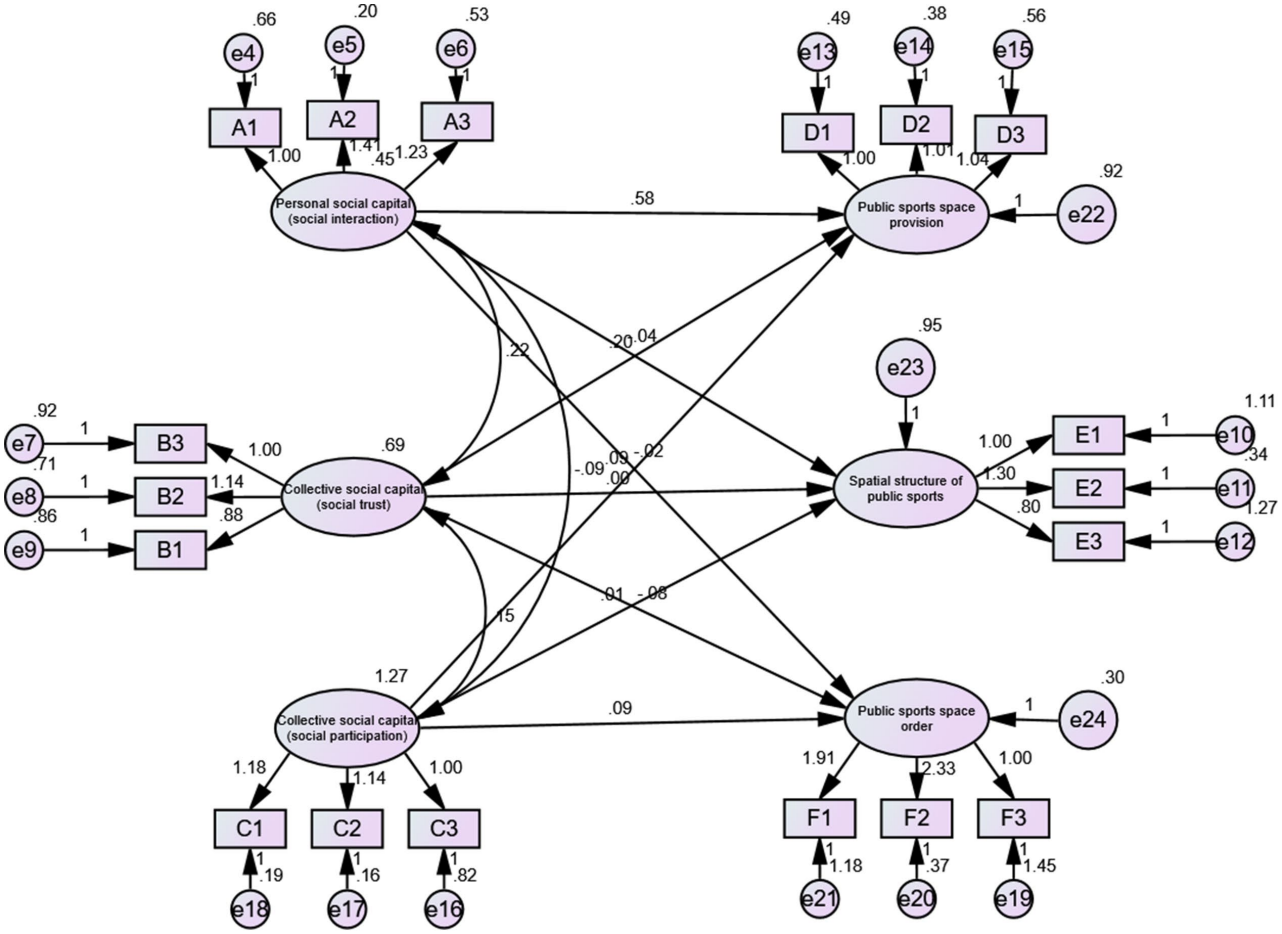
Structural equation model of social capital participation mechanism in the governance of public sports space in dilapidated urban communities.

“Collective social capital (social participation) → public sports space provision,” “collective social capital (social participation) → spatial structure of public sports,” and “collective social capital (social participation) → public sport space order “had standardized factor loadings of 0.064 (*p* = 0.205), 0.013 (*p* = 0.728), and 0.128 (*p* < 0.05) respectively; and *t*-values of 1.266, 0.276, and 2.438, respectively. Among them, only “collective social capital (social participation) → public sports space order” with |*T*| > 1.96 has a positive effect, H3c is valid, H1a and H2b are not. The standardized factor loading for “collective social capital (social participation) → governance of public sport space” was 0.024 (*p* = 0.682) and the *t*-value was 0.409 (|*T*| < 1.96), which did not have a positive effect and H3 did not hold. In summary, the standardized factor loading of “collective social capital → governance of public sport space” is 0.123 (*p* < 0.05), with a *t*-value of 2.164 (|*T*| > 1.96), possessing a positive effect, and H4 holds. In addition, the structural equation model of social capital participation mechanism in the governance of public sports space in dilapidated communities is shown in Figure 2.

## 5. Discussion

Public health governance is increasingly being driven from the top down, and building and mobilizing social capital is a crucial strategy for enhancing the management of communal public sports spaces and fostering active aging among older people. This study compares the literature on the theoretical meaning and dimensions of social capital, materialises the manifestations of social capital in terms of social interaction, social trust, and social participation, and investigates the significant role of social capital in the management of public sports spaces in historic urban areas.

Personal social capital has a beneficial impact on how public sports spaces are run in dilapidated populations. Specific public health communities foster the development of personal social capital in older populations, which can improve residents’ cross-community cooperation and foster open and inclusive social relationship network coherence. Personal social capital in managing public sports spaces in dilapidated populations is a social factor that may support their long-term autonomy in public health management (59). The successful engagement of community members, who are the primary stakeholders in the governance of public health, is an expression of their civil rights and duties and a crucial step toward enhancing their physical and mental health and quality of life (60, 61). Residents of the community are better able to make initial connections with other actors and contribute to the expansion of information channels, the reduction of information asymmetries, and the better facilitation of the exchange of public health resources when they have a wider variety of social networks (62). According to social cognitive theory, having sufficient personal social capital may improve a person’s mental, behavioural, and emotional development and increase governance efficiency in public sports spaces (63). Existing research indicates that various factors may affect how social capital manages public sports spaces in a community (64). For instance, the more highly personal values their health, the higher the level of participation in the governance of community public sports spaces; the more strongly a unique values sport, the higher the level of involvement in the government of community public sports spaces; and the higher the level of an personal’s sense of community, the higher the level of participation in the governance of community public sports spaces. The massive new crown epidemic breakout has increased community members’ demand for public health, and the management of public sports spaces in dilapidated towns is intimately tied to people’s public health concerns. Physical activity helps citizens improve their physical and mental health and social networks, successfully agree on running public sports spaces, and encourage building personal social capital. These factors all contribute to improved community governance (65). Our researchers hypothesise, based on extensive data analysis, that the likelihood of citizens participating in the administration of public sports spaces in their communities rises by 2.1% for every 10% increase in the personal social capital index (66). In conclusion, personal social capital supports the coupling of community public sports services with residents’ public health needs, strengthens residents’ emotional ties to the community, and promotes the development of a public health community by providing adequate public health resources for the governance of public sports spaces in dilapidated communities.

In dilapidated communities, the renovation of public sports spaces has a favourable impact on collective social capital. According to the collective action theory, collective social capital increases the effectiveness of collective action while lowering the cost of collective action in controlling public health (67–69). Residents in dilapidated communities can be organized and managed to cooperate to maximize the benefits of public health, even though they are occasionally inclined to shirk their duties and ride along when it comes to the governance of public sports spaces (70). This is because they share a shared interest in the governance of public sports places. The essence of collective social capital is to strengthen mutual trust between groups and expand the breadth and depth of collective action, effectively enhancing the effectiveness of public health governance. Social trust and participation already exist in the social interactions of community members (71). Collective social capital plays a significant role in resolving public sports issues in dilapidated communities and advancing the community’s public sports objectives. By actively mobilising collective social capital to improve the governance effectiveness of the available sports space in dilapidated urban communities, residents of a community who share a sense of sports governance—that is, residents who feel a sense of belonging and identification with the neighbourhood public sports space—can more easily build trust and cooperate (72–74). Collective solid social capital is vital for the governance of public sports spaces in dilapidated communities because it enables locals to work together to solve public health problems in a way that is conducive to achieving Pareto optimality, enhancing the quality of the community’s provision of public sports services, and fostering a positive environment for sports participation (75). In conclusion, collectively solid social capital can improve residents’ quality of life, control social behavior in the public sphere, encourage group activity in the community, and help create a public health community.

In conclusion, there is a direct connection between personal and social capital. On the one hand, once it is formed and mobilised, personal social capital helps all members of the community structure with issues and may be a valuable public health resource for managing public sports spaces in dilapidated communities. However, collective social capital is a form of public health property that enhances the use of public health resources by integrating human and physical resources into the administration of public sports spaces in dilapidated communities through a pluralistic network of social links. The reciprocal synergy of personal and collective social capital balances personal and communal interests, decreases public health resource waste, fosters group action, and enhances the governance of public sports spaces in dilapidated populations.

## 6. Conclusions, limitations, and recommendations

### 6.1. Conclusion

This study empirically investigates the processes of the role of social capital in the governance of public sports spaces in older adult populations based on the current state of governance of such spaces. First, Social interaction has a positive contribution to the improvement of public sports space supply in dilapidated communities. It demonstrates the link between community social engagement and control over public sports spaces in dilapidated communities. Residents of a community who connect more frequently have more robust social networks, more accessible public health resource exchanges, and greater availability of public sports spaces. However, the impact of social interaction on the stability of order in dilapidated communities and the optimization of the spatial organization of public sports is minimal. Social interaction has yet to affect the dimensions of public sports spatial structure and order in dilapidated communities, likely because social capital is stratified and public health resources are in lower strata, leading to the weakness of personal social capital in dilapidated communities.

Second, social trust has a positive contribution to the optimization of public sports space supply in dilapidated communities; this relationship shows that the higher the level of trust among community members, the higher the rate at which public health resources are converted, the stronger the community cohesion, and the more favorable the environment is for the improvement of the provision of public sports spaces. However, the optimization of the structure of public sports spaces and the stability of order in established communities are relatively unaffected by social trust. It might be because, as a result of market-oriented reform, there are more strangers living in dilapidated communities, residents are forced to interact less with one another and develop a certain distance, and social trust is low, which causes a gradual decline in the quantity and quality of collective social capital. Social trust does not affect the degree of structure and order in dilapidated communities’ public sports spaces.

Thirdly, social participation has a positive contribution to the stability of public sports space order in dilapidated communities, demonstrating that the more deeply a community’s residents engage in social activities, the more their sense of identity and community will be enhanced and the more consciously they will adhere to community rules and regulations, thereby deepening the development of public health communities and strengthening the stability of the structure of public sports spaces in communities. However, the role of social participation in improving the supply and stabilizing the structure of public sports space in dilapidated communities is not significant. It may be because the government has long led and supported the development of dilapidated communities in China, the organizational framework and system for community autonomy are less ideal, community committees and property management firms are still unable to fully take into account the universality and characteristics of residents in the governance of public sports spaces in dilapidated communities, and they do not play a role in providing timely feedback to the public. A fine-tuned approach to the public health needs of vulnerable groups within the community is needed.

### 6.2. Limitations

It is difficult to rule out the possibility of reverse causality and explain the multiple causal relationships between variables in this study because it is based on a cross-sectional study, which makes it impossible to determine the causal relationship between social capital and the governance of public sports spaces in dilapidated communities. However, social capital may have an inhibitory effect on the management of public sports spaces in dilapidated urban communities. The precision of the research’s findings might be increased by conducting a more in-depth longitudinal study to elucidate the processes of social capital engagement in the administration of public sports spaces in dilapidated populations.

The age range of the study sample was primarily concentrated between 51 and 60 years old, which tends to make it challenging to use the demographic characteristics effectively and may have some impact on the social capital participation mechanism in the governance of public sports spaces in dilapidated communities. In the future, the research population’s sample size may be suitably increased to improve the application of the demographic data, and the link between control and latent variables can be clarified to enhance the applicability of the study’s findings.

Information on the household incomes of community members has not yet been gathered for this article. According to several research, income level can influence the development and mobilisation of social capital to some extent. Therefore, to increase the validity of the results, future studies should include the inhabitants’ income level (as determined by Chinese taxation rules) as a control variable in the regression analysis.

The subject group is restricted by funding and only studied the dilapidated communities in Yue Lu District, Changsha City, Hunan Province, which are susceptible to certain contingencies due to geographical, environmental, cultural, and institutional factors and are likely to influence the research findings to some extent. These limitations make the research area for this paper somewhat restricted. To lessen the likelihood of the research findings, the research field might be broadened to examine the social capital’s involvement mechanism in managing public sports spaces in historic towns under various geographic situations.

### 6.3. Recommendations

Firstly, cultivating public spirit and reshaping the concept of sports governance. In order to establish a common and acceptable view of sports across the community, it is first crucial to incubate and foster those governance actors who are unfamiliar with public sports through publicity. Furthermore, to increase the influence of sports social organizations, enhance their management, and encourage the development of public sportsmanship. Moreover, to successfully improve the quality of the community’s provision of public sports spaces, the government should take the initiative to foster social trust, aggressively develop the spirit of public sports, and amass social capital.

Secondly, proper decentralisation and empowerment to strengthen the governance structure of the sport. To begin with, the government should modestly decentralize and pay attention to the division of responsibilities to maintain consistency in the powers and obligations of grassroots community sports organizations and to ultimately stimulate a sense of subjectivity. Community sports organizations’ professionalism and authority should be strengthened by introducing composite sports talents. The design of public sports spaces in communities should be optimized using digital technologies. In order to balance the discourse gap between the government, community, and residents caused by the power gap and to strengthen the structure of the public sports space in the community at the level of social capital, for instance, internet platforms can be used to gather feedback on the opinions of the sports public.

Thirdly, we should strengthen communication and collaboration to build an order in sports governance. Social capital is highly scarce from the standpoint of resource allocation in dilapidated communities, which weakens the dynamics of sustainable development of public sports space governance in dilapidated communities. Therefore, a communicative and collaborative strategy can strengthen sports governance’s potential. On the one hand, we continue to integrate, enhance, and hone the spatial arrangement of public sports in the community through dialogue while developing a platform for community sports governance with equal communication and collaboration. The external programmatic norms are used as the primary guide to refine the programmatic norms into a practical community public sports spatial order following the community’s current situation of sports governance. On the other hand, we provide feedback on the opinions of multiple subjects on sports governance through field interviews and questionnaires and take the external programmatic norms as the primary guide.

Additionally, public sports spaces in dilapidated metropolitan communities are threatened by physical and social degradation as a result of institutional changes brought on by rapid urbanization and the commodification of housing. Examples include the erosion of the local athletic landscape and the community’s diverse and ageing population. The trend toward weakening public sports space administration efficiency in dilapidated communities is delayed, however, by the fact that these dilapidated communities typically have solid links with the public health system and community cohesion. In addition to offering some level of protection for inhabitants’ quality of life and general health, social capital also makes up for shortcomings in the administration of public sports spaces in dilapidated communities. Theoretical and practical issues that need to be resolved and researched in the future relate to how to efficiently integrate public health resources, create a “government-market-resident” multi-governance mechanism, actively mobilize and accumulate social capital, strengthen the health promotion of older people in the community, and build a public health community.

## Data availability statement

The raw data supporting the conclusions of this article will be made available by the authors, without undue reservation.

## Ethics statement

The studies involving human participants were reviewed and approved by Hunan University of Science and Technology Ethics Committee. The patients/participants provided their written informed consent to participate in this study. Written informed consent was obtained from the individual(s) for the publication of any potentially identifiable images or data included in this article.

## Author contributions

LX is in charge of writing the thesis. ZX is in charge of supervision and guidance. HQ was responsible for adjusting the framework of the paper. All authors contributed to the article and approved the submitted version.

## Conflict of interest

The authors declare that the research was conducted in the absence of any commercial or financial relationships that could be construed as a potential conflict of interest.

## Publisher’s note

All claims expressed in this article are solely those of the authors and do not necessarily represent those of their affiliated organizations, or those of the publisher, the editors and the reviewers. Any product that may be evaluated in this article, or claim that may be made by its manufacturer, is not guaranteed or endorsed by the publisher.
